# P28 Antimicrobial activity of imipenem/relebactam and comparators against clinical Gram-negative bacilli collected in the UK: results from the Study for Monitoring Antimicrobial Resistance Trends (SMART) 2020–24

**DOI:** 10.1093/jacamr/dlag102.034

**Published:** 2026-06-26

**Authors:** M Wise, M Allen, F Siddiqui, K Young, M Motyl, D Sahm

**Affiliations:** IHMA, Schaumburg, IL, USA; MSD (UK) Limited, London, UK; Merck & Co. Inc., Rahway, NJ, USA; Merck & Co. Inc., Rahway, NJ, USA; Merck & Co. Inc., Rahway, NJ, USA; IHMA, Schaumburg, IL, USA

## Abstract

**Background:**

Imipenem/relebactam (IMI/REL) is a combination of the carbapenem, imipenem, with the β-lactamase inhibitor relebactam, an inhibitor of Class A & C β-lactamases. IMI/REL is approved in the UK and EU for adults with hospital-acquired and ventilator-associated bacterial pneumonia, as well as complicated urinary tract and complicated intra-abdominal infections. In the USA, IMI/REL is additionally approved in paediatric patients for the indications described above. We evaluated the activity of IMI/REL against isolates of non-*Morganellaceae* Enterobacterales (NME) and *Pseudomonas aeruginosa* collected in the UK as part of the global SMART surveillance program.

**Methods:**

In 2020-2024, 6 clinical laboratories in the UK each collected up to 250 consecutive, aerobic or facultative, Gram-negative pathogens per year from patients with bloodstream, intraabdominal, urinary tract, and lower respiratory tract infections. MICs were determined using the reference broth microdilution methods and interpreted with 2025 EUCAST breakpoints. Only NME were examined since members of the *Morganellaceae* family display intrinsic reduced susceptibility to imipenem by a mechanism other than β-lactamase production and relebactam does not influence imipenem susceptibility in this family. For the NME, an MDR phenotype was defined as resistance (by EUCAST criteria) to ≥3 sentinel agents (amikacin, cefepime, colistin, levofloxacin, meropenem and piperacillin/tazobactam). For *P. aeruginosa*, MDR was defined as an isolate testing as resistant (EUCAST) to ≥3 sentinel drugs: amikacin, aztreonam, cefepime, colistin, meropenem, levofloxacin and piperacillin/tazobactam. Difficult-to-treat resistant (DTR) *P. aeruginosa* was defined as an isolate testing resistant by EUCAST to all β-lactams (including aztreonam, cefepime, ceftazidime, imipenem, meropenem, piperacillin/tazobactam) and fluoroquinolones (levofloxacin). Most isolates that were imipenem-non-susceptible (2020-2022), imipenem-resistant (2023-2024) or IMI/REL-NS (2020-2024) by CLSI criteria were screened for β-lactamase carriage by PCR or whole genome sequencing.

**Results:**

A total of 3232 NME and 786 *P. aeruginosa* isolates were collected in the UK (2020-2024). Overall, 99.1% of the NME were susceptible to IMI/REL by the EUCAST breakpoint. Ceftazidime/avibactam, meropenem, imipenem, ertapenem and amikacin also inhibited >97% of the NME isolates at their respective EUCAST susceptible breakpoints. IMI/REL was able to inhibit 99.7% and 97.5% of the *Escherichia coli* and *Klebsiella pneumoniae*, respectively. Regarding isolates that were characterized molecularly, seven NME from the UK were found to carry KPC and all proved susceptible to IMI/REL. Among the *P. aeruginosa*, 96.2% of the full collection were susceptible to IMI/REL. Ceftazidime/avibactam, amikacin and colistin were also highly active, each inhibiting >96% of the population, as well. IMI/REL retained activity against 72.4%, 78.2% and 71.8% of the cefepime-, ceftazidime- and piperacillin/tazobactam-resistant isolates, respectively, approximately two to seven percentage points higher than ceftazidime/avibactam in each case. IMI/REL inhibited 71.6% of the MDR *P. aeruginosa,* five percentage points more than ceftazidime/avibactam, while both IMI/REL and ceftazidime/avibactam inhibited 25% of the *P. aeruginosa* DTR isolates.

**Conclusions:**

Based on these *in vitro* data, IMI/REL provides an important treatment option for patients in the UK with infections caused by Gram-negative pathogens, including KPC-carrying NME and most *P. aeruginosa* non-susceptible to standard-of-care antimicrobials.

